# Expression of IL-13Rα2 and FUS in glioma: clinicopathological and prognostic correlation

**DOI:** 10.1186/s12883-023-03237-z

**Published:** 2023-05-08

**Authors:** Guang Cheng, Meng Wang, Xiyue Zhang, Yun Zhang

**Affiliations:** 1 Department of Neurosurgery, Xijing Hospital, Air Force Medical University, Xi’an, China; 2 Department of Immunology, Basic Medicine School, Air Force Medical University, Xi’an, China; 3 Department of Immunology, Medicine School, Yan’an University, Yan’an, China; 4 Department of Pathogenic Biology, Medicine School, Yan’an University, Yan’an, China

**Keywords:** Glioma, IL-13Rα2, Fused in sarcoma (FUS), LGG, HGG

## Abstract

**Background:**

IL-13Rα2 is one of the most widely studied tumor-associated antigens in glioma research. Fused in sarcoma (FUS) is a DNA/RNA binding protein that is dysfunctional in various malignant tumors. However, the expression of IL-13Rα2 and FUS, their relationship with clinicopathological parameters and their prognostic value in glioma remain unclear.

**Methods:**

In the present study, the expression of IL-13Rα2 and FUS was measured in a glioma tissue array by immunohistochemistry. Pearson’s X^2^ test was used to determine the correlation between immunohistochemical expressions and clinicopathological parameters. Pearson’s or Spearman's correlation test was used to determine the association between these two proteins expression. The Kaplan–Meier analysis was used to investigate the effect of these proteins on prognosis.

**Results:**

The expressions of IL-13Rα2 were significantly higher in high-grade gliomas (HGG) than that in low-grade gliomas (LGG) and was associated with IDH mutation status, whereas FUS location demonstrated no significant correlation with clinicopathological parameters. Moreover, a positive relationship was found between nuclear and cytoplasmic co-localization FUS and IL-13Rα2 expression. Kaplan–Meier analysis revealed that patients with IDH wide type or IL-13Rα2 had worst overall survival (OS) compared to other biomarkers. In HGG, IL-13Rα2 combined with nuclear and cytoplasmic co-localization of FUS was associated with worse OS. Multivariate analysis showed that tumor grade, Ki-67, P53 and IL-13Rα2 could be the independent prognostic factors for OS.

**Conclusion:**

IL-13Rα2 expression was significantly associated with cytoplasmic distribution of FUS in human glioma samples and could be the independent prognostic factors for OS, while the prognostic value of its co-expression with cytoplasmic FUS in glioma need to be addressed in the future studies.

## Background

Gliomas are the most common malignancy in the brain and represent approximately 80% of malignant brain and other central nervous system (CNS) tumors [1]. Based on the World Health Organization (WHO) classification, gliomas are graded on a scale of I to IV [2]. In 2016, for the first time, the WHO CNS classification used molecular markers to classify gliomas, and in 2021, the fifth edition of the WHO Classification of Tumors of the Central Nervous System (WHO CNS5) became the current international standard for glioma nomenclature and diagnosis [3, 4]. In this classification system, the primary genetic markers for gliomas are IDH mutation status, codeletion of chromosomal arms 1p and 19q (1p/19q codeletion), nuclear alpha-thalassemia/mental retardation X-linked syndrome (ATRX) gene mutations, O^6^-methylguanine-DNA methyltransferase (MGMT) promoter methylation status, loss of cyclin-dependent kinase inhibitor 2A (CDKN2A), epidermal growth factor receptor (EGFR) amplification and telomerase reverse transcriptase (TERT) promoter mutations. However, gliomas are histologically and molecularly diverse, exhibiting heterogeneity both between patients and within individual tumors. The understanding of the significance biomarkers in glioma tumors is far from enough.

Specifically, gliomas are separated into two classes according to the mutation status of isocitrate dehydrogenase (IDH), which encodes the cytosolic isoform of IDH that participates in cellular respiration [5]. In glioma, the mutations occur at the arginine residue at codon 132 in IDH1 (IDH1R132H) and at codon 140 in IDH2, and since the IDH1R132H alteration accounts for 90% of IDH mutations, immunohistochemistry (IHC) evaluation with an IDH1R132H antibody can cover 90% cases of IDH1/2 mutation [6]. Besides, ATRX is often detected in the IDH-mutant group [7–9]. P53 is a tumor suppressor and has been determined to be a possible predictive and prognostic factor in gliomas [10]. In addition, IDH-mutant astrocytoma often has P53 and ATRX mutations and is often the ALT phenotype, while IDH-mutant and 1p/19q-codeleted oligodendrogliomas often have wild-type P53 and TERT promoter mutations, which are indicative of telomerase activation [11]. MGMT promoter methylation is also a molecular biomarker of glioma subtypes that has clinical applications due to its prognostic and predictive value[12–16]. These genetic layers driven a newfound appreciation for intra-tumoral heterogeneity found in gliomas. However, these biomarkers are still not sufficient to explain why suitably genotype-targeted therapies have not been successful for glioma.

IL-13Rα2 is one of the most widely studied tumor-associated antigens in glioma research, which is also overexpressed in a variety of solid cancers, including gliomas, melanoma and pancreatic, ovarian, breast, colon and prostate cancers [17, 18]. It is well known that IL-13Rα2 is overexpressed by high-grade gliomas (HGG), but not expressed at significant levels by low-grade gliomas (LGG) or normal brain tissue[19]. Over 30 variations of treatments focused on IL-13Rα2 have been used to target and kill glioma cells in vitro, and in preclinical clinical settings [20, 21]. One of the challenges of successful IL-13Rα2 target therapy, is the loss of a targeted antigen with treatment and intra-glioma heterogeneity [22]. Therefore, the expression pattern of IL-13Rα2 in glioma remains to be elucidated.

Fused in sarcoma (FUS) is a DNA/RNA binding protein involved in RNA metabolism and DNA repair [23, 24]. Numerous reports have demonstrated by pathological and genetic analysis that FUS is associated with a variety of malignant tumors and neurodegenerative diseases [25–27]. Recent studies have shown that FUS plays a crucial role in promoting the malignant progression of glioma cells [28]. In most cell types, FUS is present in both the nucleus and cytoplasm; however, in neurons, proportionally more FUS is expressed in the nucleus, while its expression in glia is exclusively nuclear [29]. Further studies have shown that normal FUS proteins are mainly located in the nucleus, whereas mutant proteins are primarily found in the cytoplasm [30, 31]. During stress, excess FUS in the cytoplasm is involved in the formation of stress granules (SGs), which are composed of mRNAs, ribosome translation initiation factors, and other RNA-binding proteins [32, 33]. According to the database for RNA binding proteins (RBP) (https://starbase.sysu.edu.cn/), IL-13Rα2 is the mRNA target of FUS. Nevertheless, whether the association between IL-13Rα2 and FUS has clinical significance in human gliomas remains unclear.

In this study, we evaluated that the expression of IL-13Rα2 and FUS in 48 clinical glioma specimens, preliminarily analyzed their relationship with clinicopathological parameters and performed a survival analysis to further infer their value as biomarkers that have prognostic, predictive, and clinical applications in glioma subtypes.

## Materials and methods

### Reagents and tissue specimens

Anti-human IL-13Rα2 (PA5-46,976; 5 μg/mL) and FUS (PA5-96,477; 1:100) antibodies were both purchased from Thermo Fisher Scientifc (Waltham, MA, USA). Tissue microarrays (TMAs) were constructed by Servicebio technology (G6042-1, Wuhan, China) using formalin-fixed paraffin-embedded blocks of surgically resected glioma specimens. Patients were selected with survival > 30 days, indicating that the patient survived from the initial surgery and radiation treatments. Additionally, data for clinicopathological biomarkers were also provided by Servicebio technology, such as Ki-67 tested by immunohistochemistry (IHC) and threshold of 30% was chosen to stratify Ki67 [34]; mutation of P53, IDH and ATRX tested by fluorescence in situ hybridization (FISH); MGMT methylation tested by Methylation-Specific PCR (MS-PCR). The characteristics of all glioma cases were also provided by Servicebio technology (Table 1). In this study, we comprehensively characterize the phenotype of 48 glial tumors resected from patients between 8 and 75 years of age. All histologically classified tumors were defined according to the WHO 2016 classification of tumors of the central nervous system. Grade 2 and 3 gliomas were referred to as LGG, whereas the grade 4 gliomas as HGG. Among the 48 gliomas cases, there were 22 LGG and 26 HGG.

**Table 1:** Characteristics of enrolled subjects

Characteristics	Number of patients
Age	
< 50	30
≥ 50	18
Gender	
Male	31
Female	17
WHO grade	
2	13
3	9
4	26

### Scoring of immunostaining

IHC results were scored via semiquantitative analysis as described by Guichet [35]. In particular, for IL-13Rα2 and FUS, the percentage of immunopositive cells was estimated by counting the number of immunopositive cells in ten high-power (40x) fields, which were systematically randomized throughout the section. For each field, the ratio of positive cells/total number of cells was calculated (%). The mean value of the ten fields obtained from a section was considered the estimated percentage of immunopositivity assigned to the tumor sample. Each case was evaluated by one person and was subsequently reviewed by a second observer and any disagreement was resolved with a third pathologist in order to achieve an ultimate decision. The percentage of positive cells was scored using a four-tiered scale: 0 (staining absent), 1 (≤ 10%), 2 (≤ 50%) and 3 (> 50%). The intensity of staining was assessed as follows: 0 (no staining), 1 (weak intensity), 2 (moderate intensity) and 3 (strong intensity). The two scores were multiplied to obtain the staining scores (between 0 and 9) after which the sections were finally divided into the low (score 0–4) and high (score 5–9) groups.

### Statistical analysis

Significance was established with GraphPad Prism 9 software. Pearson’s X^2^ test was used to determine the correlation between immunohistochemical expression and the clinicopathologic features of patients. Pearson’s or Spearman’s correlation test was used to determine the association between the immunohistochemical expression of FUS and IL-13Rα2. Overall survival (OS) was defined as the time (months) from surgical resection to death (or the latest follow-up). Univariate and multivariate analyses were used in the survival analysis by the Kaplan–Meier method and Cox hazard regression analysis. The Kaplan–Meier method was used to analyze the clinicopathological parameters associated with the prognosis of glioma patients. The Cox hazard regression analysis was a further analysis of independent factors associated with the prognosis of glioma patients on the basis of Kaplan–Meier method. All *p* values were two-sided; *p* values less than 0.05 were considered statistically significant and those less than 0.01 were considered highly significant.

## Results

### Clinicopathological characteristics of patients

The characteristics of the 48 patients are summarized in Table 2. Our cohort consisted of patients with LGG (45.8%; 22/48) and HGG (54.2%; 26/48). 31 males and 17 females with an average age of 46 years (range from 8 to 75 years) comprised the cohort. Ki-67 positivity was used to determine the 2 classes with the following percentage of positive cells: < 30% and ≥ 30%. Thus, among the 48 patients, 10 were considered class 1 (< 30%) and 38 were considered class 2 (≥ 30%). P53 positivity was also used to differentiate 2 classes: < 5% (21, 43.8%) and ≥ 5% (27, 56.2%). Cases were classified into wild-type and mutated groups based on IDH and ATRX status, and MGMT was determined to be methylated or not methylated. The characteristics of the cases were as follows: IDH mutation (18, 37.5%), ATRX mutation (27, 56.2%) and MGMT methylation (14, 29.2%). The median OS was 20.4 months (range from 1 to 47 months) and 38 patients (79.2%) died. Compared with patients with the LGG and HGG subtypes, age (*p* = 0.011), Ki-67 positivity (*p* = 0.002), p53 positivity (*p* = 0.011), and IDH mutation status (*p* = 0.001) reached statistical significance.

**Table 2:** Clinicopathological characteristics of all glioma patients

Clinicopathologic characteristics	Total (48)	LGG (Grade 2 + 3, *n* = 22)	HGG (Grade 4, *n* = 26)	*P* value
**Age**				
** < 50**	**30 (30/48, 62.5%)**	**18 (18/22, 81.8%)**	**12 (12/26, 46.2%)**	0.011^*^
** ≥ 50**	**18 (18/48, 37.5%)**	**4 (4/22, 18.2%)**	**14 (14/26, 53.8%)**	
**Gender**				
**Male**	**31 (31/48, 64.6%)**	**13 (13/22, 59.1%)**	**18 (18/26, 69.2%)**	**0.464**
**Female**	**17 (17/48, 35.4%)**	**9 (9/22, 40.9%)**	**8 (8/26, 30.8%)**	
**Ki-67**				
**< 30%**	**10 (10/48, 20.8%)**	**9 (9/22, 40.9%)**	**1 (1/26, 3.8%)**	0.002^*^
** ≥ 30%**	**38 (38/48, 79.2%)**	**13 (13/22, 59.1%)**	**25( 25/26, 96.2%)**	
**P53**				
** wt**	**21 (21/48, 43.8%)**	**14 (14/22, 63.6%)**	**7 (7/26, 26.9%)**	0.011^*^
** mut**	**27 (27/48, 56.2%)**	**8 (8/22, 36.4%)**	**19 (19/26, 73.1%)**	
**IDH1**				
** wt**	**30 (30/48, 62.5%)**	**7 (7/22, 31.8%)**	**23 (23/26, 88.5%)**	0.001^*^
** mut**	**18 (18/48, 37.5%)**	**15 (15/22, 68.2%)**	**3 (3/26, 11.5%)**	
**ATRX**				
** wt**	**21 (21/48, 43.8%)**	**8 (8/22, 36.4%)**	**13 (13/26, 50.0%)**	**0.343**
** mut**	**27 (27/48, 56.2%)**	**14 (14/22, 63.6%)**	**13 (13/26, 50.0%)**	
**MGMT methylation**				
** Negative**	**34 (34/48, 70.8%)**	**15 (15/22, 68.2%)**	**19 (19/26, 73.1%)**	**0.71**
** Positive**	**14 (14/48, 29.2%)**	**7 (7/22, 31.8%)**	**7 (7/26, 26.9%)**	

*HGG* High-grade gliomas, *LGG* Low-grade gliomas

*P* values were calculated by Pearson’s X^2^ test (two sided)

^*^P < 0.05 indicates statistical signifificance

### The expression levels of IL-13Rα2 and FUS and the association between these two protein markers

**Fig. 1:**
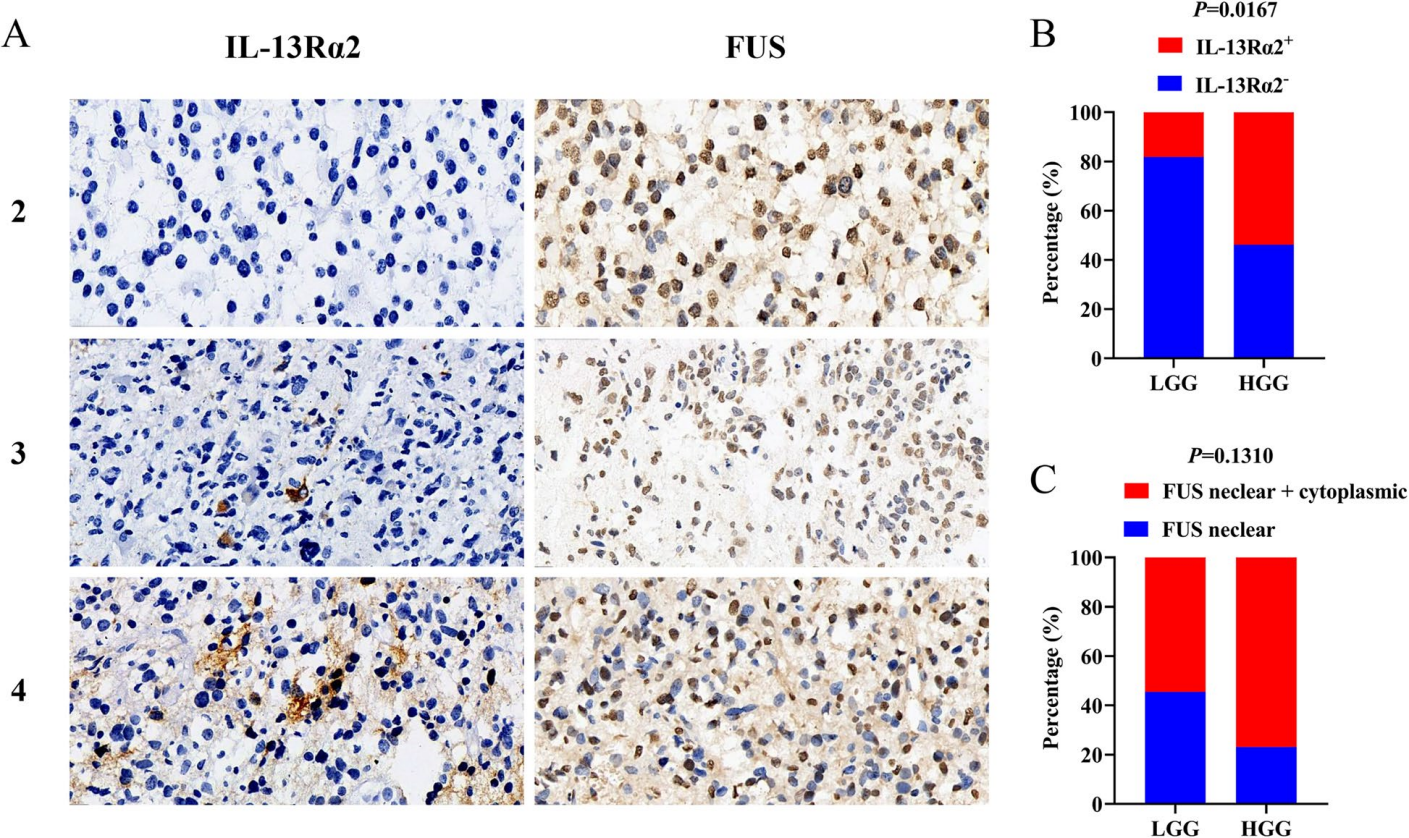
Expression of IL-13Rα2 and FUS in glioma tissues. **A** Proteins expression in glioma patients with WHO grade 2 to 4 (400 ×). **B** Expression levels of IL-13Rα2 in LGG and HGG. **C** FUS with nuclear location or both nuclear and cytoplasmic location in LGG and HGG

To assess the protein expression and status of IL-13Rα2 and FUS in clinical glioma samples, we performed immunostaining with anti-IL-13Rα2 and anti-FUS. The immunohistochemical staining showed that IL-13Rα2 was located in the cytoplasm and/or membrane in a variable number of tumor cells (Fig. 1A, left column). In Fig. 1B, we detected positive expression of IL-13Rα2 in 4 of 22 LGG cases (18.2%) and in 14 of 26 HGG cases (53.8%). In HGG, 12 positive cases showed high IL-13Rα2 staining, while in LGG, both positive cases exhibited low IL-13Rα2 staining. Statistical analysis showed that high IL-13Rα2 staining was significantly increased in HGG compared with LGG (*p* = 0.0167) and was also accompanied by strong cytoplasmic and/or membranous staining.

Our study showed that FUS was ubiquitously expressed in either the nucleus alone or in both the nucleus and the cytoplasm (Fig. 1A, right column). In Fig. 1C, of the 22 LGG cases and 26 HGG cases, 10 (45.5%) and 6 (23.1%), respectively, exhibited FUS nuclear localization, while 12 LGG cases (54.5%) and 20 HGG cases (76.9%) exhibited both FUS nuclear and cytoplasmic staining. Moreover, 12 IL-13Rα2-positive cases in the HGG group showed colocalization with FUS in the nucleus and cytoplasm, and only 1 case showed FUS nuclear localization. Additionally, 4 of 6 HGG cases with nuclear FUS expression were IL-13Rα2-negative, and only 2 cases were IL-13Rα2-positive. While, FUS nuclear and cytoplasmic staining showed no statistical significance between HGG and LGG (*p* = 0.1310).

**Fig. 2:**
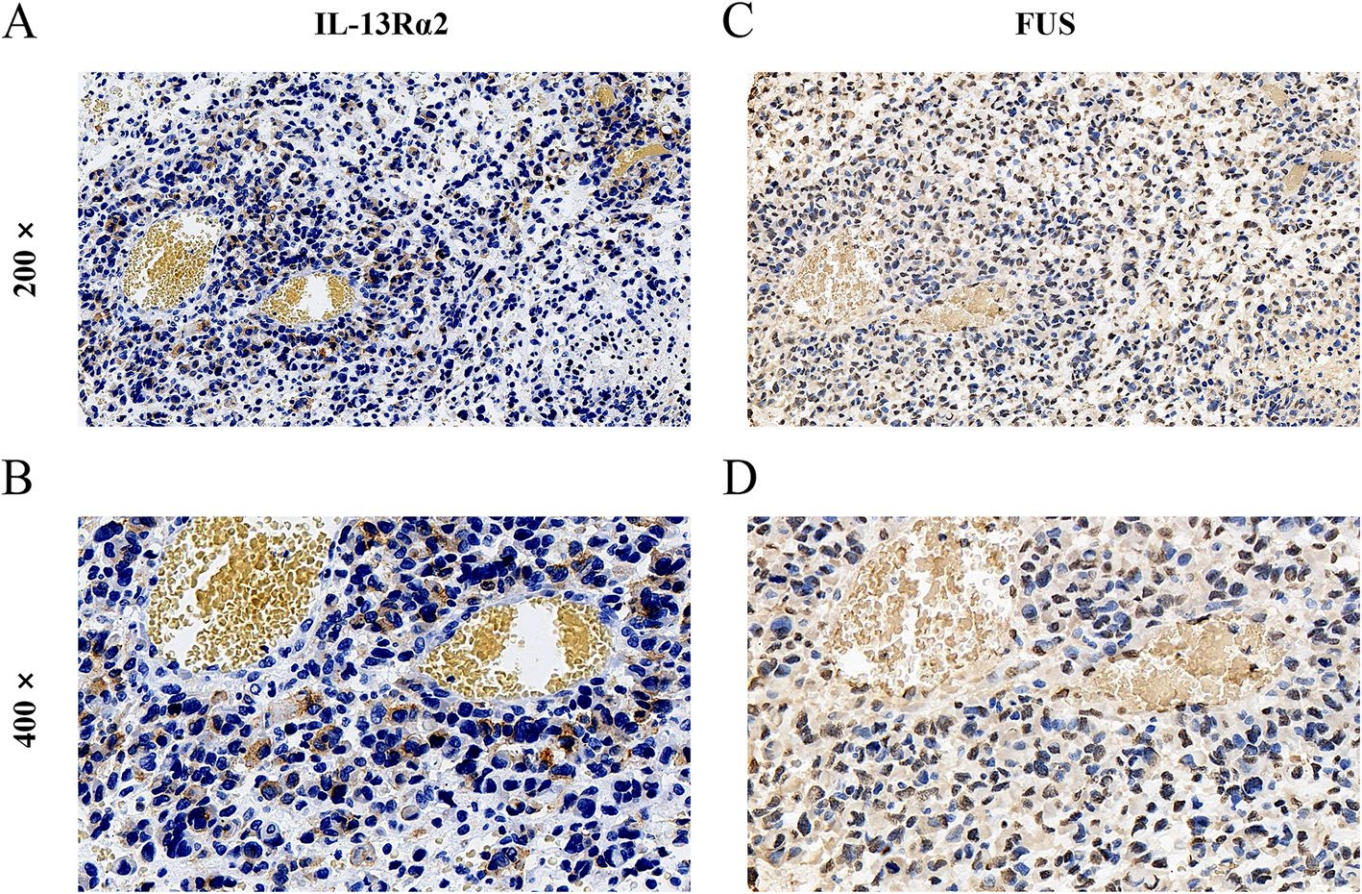
Concurrent expression of IL-13Ra2 and FUS with both nuclear and cytoplasmic location in the same samples. **A** IL-13Rα2 expression (200 ×). **B** IL-13Rα2 expression (400 ×). **C** FUS nuclear and cytoplasmic location (200 ×). **D** FUS nuclear and cytoplasmic location (400 ×)

Thus, we further performed a correlation analysis to clarify the relationship between these two proteins. Pearson’s correlation analysis revealed a positive correlation between IL-13Rα2 and cytoplasmic FUS expression (*p* = 0.0129; *r* = 0.3565) in glioma. IHC staining on continuous tissue sections further confirmed that high expression of IL-13Rα2 and cytoplasmic FUS expression were concurrent in the same samples (Fig. 2), which implies that IL-13Rα2 may cooperate with cytoplasmic FUS in gliomagenesis.

### Association between the expression levels of IL-13Rα2 and FUS and clinicopathological characteristics

We evaluated the relationship between the expression levels of IL-13Rα2 and FUS and clinicopathological parameters among glioma patients (Table 3). The IL-13Rα2 expression was related to IDH mutation status (*p* = 0.021). We did not find any relationship between different location of FUS and clinicopathological characteristics.

**Table 3:** Clinicopathological characteristics according to proteins expression of IL-13Rα2 and FUS in all glioma patients

Clinicopathologic characteristics	IL-13Rα2		*P* value	FUS		*P* value
	**negative (*n* = 30)**	**positive (*n* = 18)**		**nucleus (*n* = 16)**	**nucleus and cytoplasmic (*n* = 32)**	
**Age**						
** < 50**	**21 (70.0%)**	**9 (50.0%)**	**0.165**	**13 (81.3%)**	**18 (56.3%)**	**0.088**
** ≥ 50**	**9 (30.0%)**	**9 (50.0%)**		**3 (18.7%)**	**14 (43.7%)**	
**Gender**						
**Male**	**19 (63.3%)**	**12 (66.7%)**	**0.815**	**9 (56.3%)**	**22 (68.7%)**	**0.393**
**Female**	**11 (36.7%)**	**6 (33.3%)**		**7 (43.7%)**	**10 (31.3%)**	
**Ki-67**						
** < 30%**	**8 (26.7%)**	**2 (11.1%)**	**0.198**	**4 (25.0%)**	**5 (15.6%)**	**0.433**
** ≥ 30%**	**22 (73.3%)**	**16 (88.9%)**		**12 (75.0%)**	**27 (84.4%)**	
**P53**						
**wt**	**15 (50.0%)**	**6 (33.3%)**	**0.259**	**6 (37.5%)**	**15 (46.9%)**	**0.537**
**mut**	**15 (50.0%)**	**12 (66.7%)**		**10 (62.5%)**	**17 (53.1%)**	
**IDH1**						
**wt**	**15 (50.0%)**	**15 (83.3%)**	0.021^*^	**8 (50.0%)**	**22 (68.7%)**	**0.206**
**mut**	**15 (50.0%)**	**3 (16.7%)**		**8 (50.0%)**	**10 (31.3%)**	
**ATRX**						
**wt**	**11 (36.7%)**	**10 (55.6%)**	**0.202**	**7 (43.7%)**	**13 (40.6%)**	**0.836**
**mut**	**19 (63.3%)**	**8 (44.4%)**		**9 (56.3%)**	**19 (59.4%)**	
**MGMT methylation**						
**Negative**	**24 (80.0%)**	**10 (55.6%)**	**0.071**	**13 (81.3%)**	**22 (68.7%)**	**0.358**
**Positive**	**6 (20.0%)**	**8 (44.4%)**		**3 (20.7%)**	**10 (31.3%)**	

*P* values were calculated by Pearson’s X^2^ test (two sided)

^*^P < 0.05 indicates statistical signifificance

### Survival analysis

**Fig. 3:**
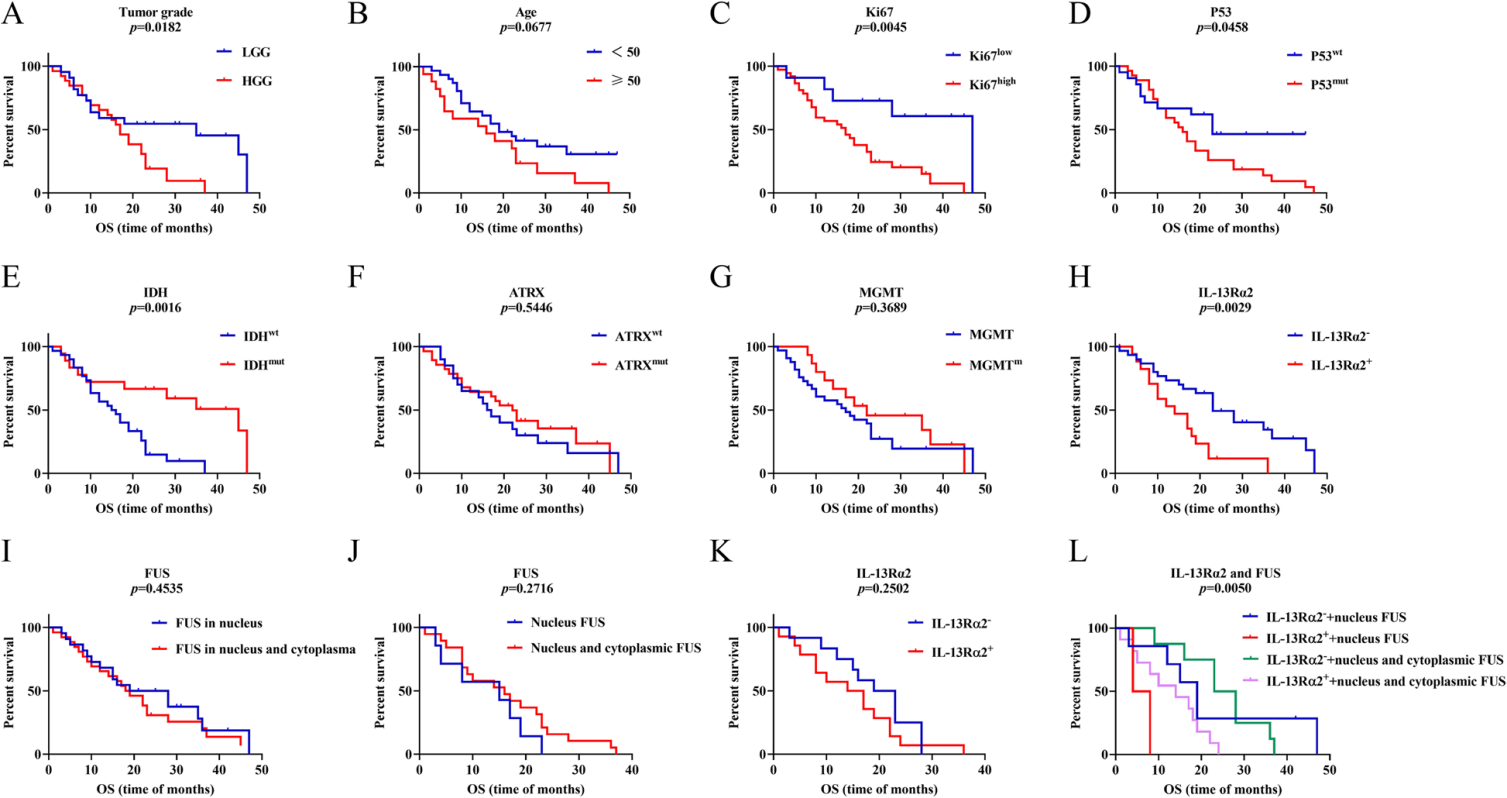
Kaplan–Meier survival analysis for OS in all glioma patients. **A** Patients with HGG had a poorer OS (*p* = 0.0182). **B** Patients with age ≥ 50 had no different OS time compared to age < 50 (*p* = 0.0677). **C** Patients with Ki-67 ≥ 30% had a poorer OS (*p* = 0.0045). **D** Patients with P53 mutation had a poorer OS (*p* = 0.0458). **E** Patients with IDH mutation had a longer OS time (*p* = 0.0016). **F**, **G** Patients with ATRX mutation (**F**) or MGMT mutation (**G**) had no different OS time compared to wide types (*p* = 0.5446 and *p* = 0.3689, respectively). **H** Patients with IL-13Rα2 expression showed worse OS (*p* = 0.0029). **I** Patients with FUS located both in nuclear and cytoplasm showed no different OS time compared to FUS located in nuclear (*p* = 0.4535). **J**, **K**, **L** Expression of IL-13Rα2 and FUS in HGG and their relationship with prognosis. Patients with FUS located both in nucleus and cytoplasm had no different OS time compared to FUS located in nuclear (*p* = 0.2716, **J**). Patients with IL-13Rα2 expression showed no different OS time compared to patients without IL-13Rα2 expression (*p* = 0.2502, **K**) and patients with IL-13Rα2 and both nuclear and cytoplasmic FUS co-expression had worst OS (*p* = 0.0050, **L**)

A survival analysis was performed for the total cohort. According to the univariate survival analysis conducted using the Kaplan‒Meier method, the following prognostic factors were found to affect OS: tumor grade (LGG vs. HGG; *p* = 0.0182), age (< 50 vs. ≥ 50 years of age; *p* = 0.0677), Ki-67 expression (< 30% vs. ≥ 30%; *p* = 0.0045), P53 status (wild-type vs. mutation; *p* = 0.0458), IDH status (wild-type vs. mutation; *p* = 0.0016), ATRX status (wild-type vs. mutation; *p* = 0.5446), MGMT methylation status (wild-type vs. methylation; *p* = 0.3689), IL-13Rα2 expression (negative vs. positive; *p* = 0.0029) and FUS expression (nuclear vs. nuclear and cytoplasmic *p* = 0.4535) (Fig. 3A-I). The results of the multivariate analysis (Cox regression analysis model) of OS that can be the independent prognostic factors or OS were as follows: tumor grade (HR 2.029; 95% CI 0.5536 to 9.059; *p* = 0.0213), IL-13Rα2 (HR 2.382; 95% CI 1.056 to 5.380; *p* = 0.0010), Ki-67 (HR 3.439; 95% CI 1.098 to 13.44; *p* = 0.0079) and P53 (HR 1.317; 95% CI 0.6046 to 3.052; *p* = 0.0113). In HGG, FUS (nuclear vs. nuclear and cytoplasmic staining) and IL-13Rα2 expression were not related to OS (*p* = 0.2716, Fig. 3J; *p* = 0.2502, Fig. 3K), and the relationship between OS and IL-13Rα2 co-expression with nuclear/cytoplasmic FUS was statistically significant (*p* = 0.0050, Fig. 3L). However, in Cox regression analysis model, expression of IL-13Rα2, FUS location or co-expression of these two molecules had no statistical significance for OS in HGG.

## Discussion

Gliomas with identical histopathological classifications may behave differently in terms of clinical outcomes and responses to treatment. The molecular patterns of glioma can partially explain clinical outcomes and predict treatment responses. Understanding the specific pathogenesis of the onset and heterogeneity of gliomas is critical for the diagnosis, and prognostic prediction in glioma patients as well as for therapy. IL-13Rα2 is the most common therapeutic target in glioma; Recent studies have shown that IL-13Rα2 can signal through the AP-1 pathway where it cooperates with other molecules, such as chitinase 3-like 1 (CHI3L1) [36] and epidermal growth factor receptor variant III (EGFRvIII), in glioblastoma [19, 37]. Studies have also reported that IL-13Rα2 stimulates human glioma cell growth and metastasis through the Src/PI3K/Akt/mTOR signaling pathway [38] Despite advances in the understanding of IL-13Rα2 biology in glioma tumors and clinical trials targeting this receptor for therapy, the regulation and prognostic significance of IL-13Rα2 expression in glioma is not well understood. As an important RNA binding protein, FUS is predicted to bind to the mRNA of IL-13Rα2. Therefore, the expression of these two molecules and their relationships with clinicopathological parameters should be addressed firstly.

In the present study, we found that IL13-Rα2 expression was much higher in HGG than that in LGG, which was consistent with the findings of previous studies. Statistical analysis showed that IL-13Rα2 expression was significantly increased in patients with IDH wide type. Next, we found that IL-13Rα2 expression had a positive relationship with the cytoplasmic FUS. These results suggested that IL13-Rα2 combined with FUS location might have prognostic value.

FUS is dysregulated in a variety of tumors types [39, 40]. Recently, FUS have reported to be upregulated in glioma tissues and cells, while downregulation of FUS significantly inhibits the malignant behavior of glioma cells, which suggests that FUS may act as an oncogene [28]. It is also well known that FUS can enter the nucleus and that mutation are associated with its cytoplasmic localization, which is associated with a variety of neurodegenerative diseases and suggests that cytoplasmic FUS might be a reliable indicator of glioma malignancy. In the present study, we found that FUS location was correlated with IL-13Rα2 expression. In univariate survival analysis, significant correlations were observed between poor OS and tumor grade, age, Ki67 and IDH but not IL-13Rα2 expression or nuclear and cytoplasmic FUS location. However, in HGG, co-expression of IL-13Rα2 and nuclear and cytoplasmic FUS was correlated with worse OS, which suggested that the combination of these two markers might be a more precise approach to predict glioma progression.

Additionally, in multivariate analysis of OS, the prognostic factors were tumor grade, Ki67, p53 and IL-13Rα2. Because the sample size was too limited in our research, the prognostic value of co-expression of IL-13Rα2 and cytoplasmic FUS should be further explored.

## Conclusions

In summary, our findings demonstrated that IL-13Rα2 expression was significantly associated with cytoplasmic distribution of FUS in human glioma samples. Expression of IL-13Rα2 may be a reliable prognostic biomarker. However, the prognostic value of co-expression of IL-13Rα2 and cytoplasmic FUS need to be addressed in the future studies.

## Data Availability

The datesets in this study are available from the corresponding author on reasonable request.

## Abbreviations

FUS: Fused in sarcoma
HGG: High-grade gliomas
LGG: Low-grade gliomas
CNS: Central nervous system
WHO CNS5: The fifth edition of the WHO Classification of Tumors of the Central Nervous System
ATRX: Alpha-thalassemia/mental retardation X-linked syndrome
MGMT: O6-methylguanine-DNA methyltransferase
CDKN2A: Cyclin-dependent kinase inhibitor 2A
EGFR: Epidermal growth factor receptor
TERT: Telomerase reverse transcriptase
IDH: Isocitrate dehydrogenase
IHC: Immunohistochemistry
FISH: Fluorescence in situ hybridization
MS-PCR: Methylation-Specific PCR
SGs: Stress granules
RBP: RNA binding proteins
TMAs: Tissue microarrays
OS: Overall survival
CHI3L1: Chitinase 3-like 1
EGFRvIII: Epidermal growth factor receptor variant III
